# Role of Complete Duplex Ultrasound to Diagnose Deep Vein Thrombosis in COVID-19 Critical Patients

**DOI:** 10.1177/1544316720985812

**Published:** 2021-03

**Authors:** Matteo Pozzi, Marco Giani, Benedetta Fumagalli, Mariangela Calabria, Davide Leni, Vittorio Segramora, Giacomo Bellani, Giuseppe Foti

**Affiliations:** 1 ASST Monza, Italy; 2 Università degli Studi di Milano-Bicocca, Monza, Italy

**Keywords:** anticoagulants, COVID-19, critical care, ultrasonography, Doppler, venous thrombosis

## Abstract

An increased risk of deep vein thrombosis (DVT) has been reported in critical COVID-19 patients, despite adequate thromboprophylaxis, and most of DVT are probably asymptomatic. As a screening approach has been advocated, the best examination protocol is unknown. The objective of this study is to assess the role of a Complete Duplex Ultrasound (CDUS) examination in detecting DVT in a large population of COVID-19 patients admitted to intensive care unit (ICU) for respiratory failure. Single-center retrospective study of 145 COVID-19 patients admitted to ICU. DVT was assessed with a CDUS performed by experienced radiologist after ICU admission. DVT was confirmed in 38 patients (26%). Most DVT were distal to the knee (66%), while only 16% were proximal. At the time of the examination, 55% of the patients received full-dose anticoagulation, while 45% received thromboprophylaxis, and there were no differences in anticoagulation regimen between positive and negative patients. Patients with DVT had higher dimers compared with those with negative CDUS (*P* < .002). The observed frequency of DVT is high despite adequate anticoagulation. A comprehensive and experienced ultrasound examination protocol can allow to recognize a large number of distal DVT otherwise missed, albeit their clinical significance is unknown.

## Background

Deep vein thrombosis (DVT) represents a serious complication in critically ill patients, accounting for a significant burden of morbidity and mortality.^1^ Ultrasonography surveillance has been proposed as a promising approach, as it allows early detection of silent DVTs. However, its ability to improve patient outcome is uncertain.^2^ When assessing lower extremity DVT, guidelines^3^ recommend a Complete Duplex Ultrasound (CDUS) protocol with Doppler rather than a limited or complete compression-only examination. The prevalence of DVT in patients with SARS-CoV-2-related respiratory failure seems higher than rates reported in historical intensive care unit (ICU) cohorts.^4-10^ As most DVT are probably asymptomatic, the value of a screening approach has been hypothesized.^11^

The aim of this study is to assess the role of CDUS in detecting DVT in a large population of COVID-19 patients admitted to ICU for respiratory failure.

## Methods

We conducted a retrospective analysis of prospectively collected data. The study included all consecutive patients with respiratory failure related to COVID-19 requiring intubation and mechanical ventilation admitted to the 5 ICUs of ASST Monza (Italy), a large academic hospital, between February 20 and April 30, 2020.

Three experienced interventional radiologists performed CDUS following clinical indication. Femoral, popliteal, and deep calf veins were screened for DVT. Complete Duplex Ultrasound protocol included B-mode compressions, color Doppler, and pulsed-wave Doppler. Deep vein thrombosis was classified into 3 categories: proximal (at the thigh), popliteal (at the knee), and distal (from knee to ankle). In patients with Internal Jugular Vein (IJV) Central Venous Catheter (CVC), the presence of jugular thrombosis was assessed in the same examination. We also collected patient comorbidities and risk factors for DVT, laboratory tests results (at ICU admission and whenever a CDUS exam was performed), incidence of computed tomography (CT) confirmed pulmonary embolism (PE) and proportion of patients who required placement of an Inferior Vena Cava (IVC) filter.

After March 21, by institutional protocol, all patients hospitalized for COVID-19 regardless of clinical severity received Low Molecular Weight Heparin (LMWH) prophylaxis and those with dimers >5000 ng/mL were fully anticoagulated with adequate LMWH or Unfractionated Heparin (UFH) dosage. Complete Duplex Ultrasound was performed after clinical decision in patients with positive D-dimers.

Data were gathered in the context of a larger prospective data collection on COVID-19 patients approved by the local Ethical Committee. Written informed consent was waived.

Data are presented as median [q1-q3] or absolute (relative) frequency. Wilcoxon’s and Fisher’s tests were used to compare numerical and categorical variables, respectively.

## Results

**Figure 1.**
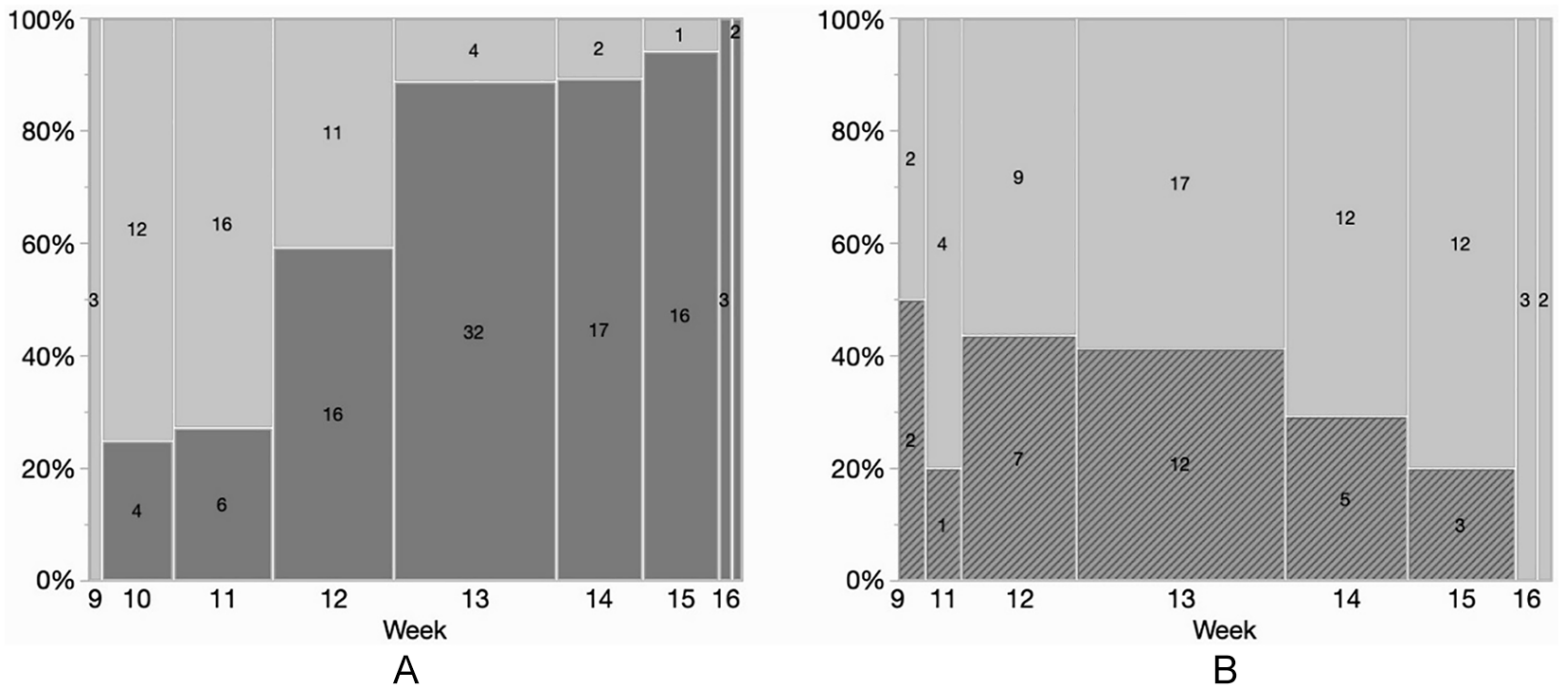
CDUS exams from week 9 (starting on February 24) to week 17 (ending on April 27). (Panel A) Number of patients screened with CDUS (dark gray). (Panel B) Number of positive (dark gray, line pattern) versus negative (light gray) CDUS. *Note.* CDUS = Complete Duplex Ultrasound.

Among 168 consecutive patients admitted to the COVID ICUs in the study period, 23 were excluded (12 patients treated with extracorporeal membrane oxygenation, 11 patients admitted for a reason other than respiratory failure). In total, 145 patients (29 females) were included in the study (see Table 1 for baseline characteristics). No patient had history of DVT or known coagulopathy, and no patient was pregnant at the time of admission. We did not record signs of DVT in any patient for the entire study duration. CDUS was performed in 96 patients (66%). Complete Duplex Ultrasound was performed 4 (1-7) days after ICU admission. During the study period, the proportion of made patients pleural screened with ultrasound progressively increased over time (Figure 1).

**Table 1.** Patient Characteristics and Laboratory Test Results at ICU Admission and at CDUS Examination.

	Patients screened with CDUS N = 96	Patient not screened CDUS N = 49	*P* value	Patients with positive CDUS N = 38	Patients with negative CDUS N = 58	*P* value
Age, years	62 [58-68]	66 [59-70]	.197	62 [59-68]	63 [57-68]	.724
BMI >30 kg/m^2^	19 (19.8%)	6 (12.2%)	.255	7 (18.4%)	12 (20.7%)	.785
Hypertension	41 (42.7%)	28 (57.1%)	.099	18 (47.4%)	23 (39.7%)	.455
Diabetes	16 (16.7%)	10 (20.4%)	.579	8 (21.1%)	8 (13.8%)	.351
Chronic heart disease	11 (11.5%)	8 (16.3%)	.411	4 (10.5%)	7 (12.1%)	.817
ICU admission						
aPTT	1.00 [0.93-1.10]	0.98 [0.93-1.1]	.393	0.99 [0.93-1.08]	1.00 [0.93-1.12]	.559
INR	1.23 [1.12-1.35]	1.26 [1.10-1.35]	.772	1.25 [1.17-1.37]	1.17 [1.11-1.35]	.105
D-dimer, ng/mL	1178 [621-6096]	935 [437-6369]	.172	2190 [872-15 243]	951 [444-3016]	.008
Fibrinogen, mg/dL	690 [484-853]	599 [435-739]	.069	653 [408-853]	705 [532-851]	.532
CRP, mg/dL	18.9 [10.7-25.6]	16.9 [10.6-27.6]	.950	20.7 [13.5-27.2]	17.8 [8.9-24.7]	.161
CDUS examination						
Days from ICU admission at 1st CDUS	4 [1-7]	—		4 [1-8]	4 [2-6]	.632
aPTT	1.05 [0.97-1.23]	—		1.03 [0.95-1.22]	1.06 [0.99-1.24]	.555
INR	1.12 [1.04-1.21]	—		1.14 [1.04-1.28]	1.12 [1.04-1.20]	.608
D-dimer, ng/mL	1881 [912-4155]	—		2573 [1514-6271]	1348 [521-2607]	.002
Fibrinogen, mg/dL	574 [344-771]	—		553 [340-766]	581 [345-798]	.571
CRP, mg/dL	9.4 [3.6-19.2]	—		12.4 [4.0-16.3]	9.3 [3.5-21.4]	.982
Anticoagulation						
UFH (IU/die)						
Full-dose	26 400 [24 000-33 600]			29 880 [24 000-33 600]	24 000 [20 000-28 800]	.131
Prophylaxis	15 000 [10 020-19 150]			15 000 [10 080-17 500]	15 000 [10 000-19 200]	.824
LMWH (IU/die)						
Full-dose	12 000 [12 000-16 000]			12 000 [12 000-20 000]	12 000 [12 000-16 000]	.903
Prophylaxis	6000 [6000-8000]			6000 [6000-6000]	6000 [6000-8000]	.559

*Note.* ICU = intensive care unit; CDUS, Complete Duplex Ultrasound; BMI, Body Mass Index; aPTT, Activated Partial Thromboplastin Time; INR, International Normalized Ratio; CRP, C Reactive Protein; UFH, Unfractioned Heparin; LMWH, Low Molecular Weight Heparin.

Deep vein thrombosis was confirmed in 38 patients (26% of the study population, 40% of the screened patients). Most of DVTs were distal (62%), whereas 22% were diagnosed at the knee (popliteal veins) and 16% were proximal. In 2 patients distal DVT progressed to proximal sites (popliteal vein, femoral vein) despite full anticoagulation.

At the time of the first CDUS, 55% of patients were already receiving full-dose anticoagulation, while 45% were receiving prophylaxis (see Table 1 for heparin dosage).

Among screened patients, those with DVT had significantly higher dimers compared with those with a negative CDUS, both at ICU admission and at CDUS examination (Table 1).

Catheter-related IJV thrombosis was diagnosed in 9 patients. Nineteen cases of PE were recorded. Pulmonary embolism occurred in 23.7% of patients with a positive CDUS and in 13.8% of patients with a negative CDUS (*P* = .214). Twelve patients required placement of an IVC filter.

## Discussion

In this cohort study on COVID-19 patients requiring mechanical ventilation for respiratory failure, we report a high frequency (26%) of asymptomatic DVT despite anticoagulation. This is consistent with previous reports in COVID-19 patients^4,5,7,8,10^ and confirms the clinical relevance of this phenomenon, if we consider that, at the time of the first CDUS exam, more than half of patients were receiving a full-dose anticoagulation. Such a disproportionate failure rate suggests that traditional DVT prophylaxis may be inadequate to face the profound pro-inflammatory and pro-thrombotic profile that characterizes COVID-19.

However, the implementation of an aggressive anticoagulation protocol, driven by the clinical evidence of this increased thrombotic risk based on early reports,^7,12^ may account for the lower incidence of thrombotic events compared with that reported from China at the beginning of the pandemic period.^4,10^

Our results confirm a significant rate of infra-popliteal thrombosis in COVID-19 ICU patients (up to 95%),^4,6,7^ which would have been missed with a standard compressive ultrasonographic study on proximal veins, and underlines the pivotal role of a comprehensive evaluation of the entire venous district performed by an experienced operator. The role and the real hazard related to distal DVT in critically ill patients is unclear, but a progression to proximal DVT was observed in 2 patients despite full anticoagulation.

Our study has 2 main limitations. First, we did not perform a rigorous screening campaign of all patients admitted to ICU. At ICU admission, patients who were screened with CDUS had similar characteristics with those who were not screened. A significant proportion of patients in the latter group had high d-dimer levels (one quarter of patients over 6300 ng/ml). Therefore, we cannot exclude that a significant proportion of patients who were not screened with CDUS had undiagnosed DVT. This circumstance was related to resource allocation facing a disproportionated and mounting number of patients.

Second, we did not perform a follow-up examination of patient after ICU discharge. This prevented us from detecting recurrent DVTs after the resolution of the first episode and those DVTs that would occur later in the clinical course of this illness.

Further studies are needed to elucidate the exact incidence of thrombotic events in the acute and chronic phase of COVID-19 disease.
